# Associated bacterial communities, confrontation studies, and comparative genomics reveal important interactions between *Morchella* with *Pseudomonas* spp.

**DOI:** 10.3389/ffunb.2023.1285531

**Published:** 2023-12-13

**Authors:** Guillaume Cailleau, Buck T. Hanson, Melissa Cravero, Sami Zhioua, Patrick Hilpish, Celia Ruiz, Aaron J. Robinson, Julia M. Kelliher, Demosthenes Morales, La Verne Gallegos-Graves, Gregory Bonito, Patrick S.G. Chain, Saskia Bindschedler, Pilar Junier

**Affiliations:** ^1^ Laboratory of Microbiology, University of Neuchâtel, Neuchâtel, Switzerland; ^2^ Bioscience Division, Los Alamos National Laboratory, Los Alamos, NM, United States; ^3^ Center for Integrated Nanotechnologies, Los Alamos National Laboratory, Los Alamos, NM, United States; ^4^ Department of Plant, Soil and Microbial Sciences, Michigan State University, East Lansing, MI, United States

**Keywords:** bacteriome, morel, fruiting body, mycelium, sclerotia

## Abstract

Members of the fungal genus *Morchella* are widely known for their important ecological roles and significant economic value. In this study, we used amplicon and genome sequencing to characterize bacterial communities associated with sexual fruiting bodies from wild specimens, as well as vegetative mycelium and sclerotia obtained from *Morchella* isolates grown *in vitro*. These investigations included diverse representatives from both Elata and Esculenta *Morchella* clades. Unique bacterial community compositions were observed across the various structures examined, both within and across individual *Morchella* isolates or specimens. However, specific bacterial taxa were frequently detected in association with certain structures, providing support for an associated core bacterial community. Bacteria from the genus *Pseudomonas* and *Ralstonia* constituted the core bacterial associates of *Morchella* mycelia and sclerotia, while other genera (e.g., *Pedobacter* spp., *Deviosa* spp., and *Bradyrhizobium* spp.) constituted the core bacterial community of fruiting bodies. Furthermore, the importance of *Pseudomonas* as a key member of the bacteriome was supported by the isolation of several *Pseudomonas* strains from mycelia during *in vitro* cultivation. Four of the six mycelial-derived *Pseudomonas* isolates shared 16S rDNA sequence identity with amplicon sequences recovered directly from the examined fungal structures. Distinct interaction phenotypes (antagonistic or neutral) were observed in confrontation assays between these bacteria and various *Morchella* isolates. Genome sequences obtained from these *Pseudomonas* isolates revealed intriguing differences in gene content and annotated functions, specifically with respect to toxin-antitoxin systems, cell adhesion, chitinases, and insecticidal toxins. These genetic differences correlated with the interaction phenotypes. This study provides evidence that *Pseudomonas* spp. are frequently associated with *Morchella* and these associations may greatly impact fungal physiology.

## 1 Introduction

*Morchella* is an iconic and highly diverse fungal genus in the Ascomycota phylum. Recent phylogenies based on multilocus molecular analyses suggest that the genus comprises at least 80 phylogenetically distinct species (Loizides et al., 2022), that can be grouped into three clades: Elata (black morels), Esculenta (yellow morels), and Rufobrunnea (grey morels) (O'Donnell et al., 2011). The fruiting bodies (ascocarps or fructifications) of *Morchella*, known commonly as morels, are highly prized for their gustative qualities and are of great economic and cultural importance (Xu et al., 2022). Wild morels are collected and exported intensively in China, India, Pakistan, Turkey, and North America (Liu et al., 2018). In recent years, outdoor morel cultivation has become successful in China, where the land area used to cultivate morels has increased from 300 to more than 10,000 ha after 2018 (Xu et al., 2022; Liu et al., 2023). The diversity of *Morchella* spp. in China is reported to correspond to close to 30 phylogenetic species. From those, only 3 to 7 are considered to be easy to cultivate under controlled condition, with the species *Morchella importuna, Morchella sextelata*, and *Morchella eximia* as the most commonly cultivated species currently (Liu et al., 2018; Liu et al., 2023). However, ascocarp production remains highly variable, which is thought to result from the complex life cycle and ecology of these emblematic fungi (Du et al., 2016; Li et al., 2017).

Many abiotic factors, such as temperature, humidity and nutrient availability, are known to influence fruiting body formation (Pilz et al., 2007). In natural habitats, nutrient availability is known to improve after wildfires, which lead to the fruiting of some species of post-fire *Morchella* (Li et al., 2017). Regarding the biotic factors affecting fruiting body production and the overall lifecycle of *Morchella* spp. in the wild, the interaction with other soil microorganisms is thought to influence the growth and development of mature ascocarps. It has been hypothesized that fungal-associated bacteria, and in particular *Pseudomonas* spp., may stimulate primordia differentiation, a key stage in the formation of fruiting bodies (Li et al., 2017). A similar effect has been suggested for black and white truffles, another group of phylogenetically related ascomycetes with subterranean fruiting bodies. In these fungi, bacteria are thought to play a role in the development, maturation, and even the final aroma of the mature fruiting body (Antony-Babu et al., 2014; Splivallo et al., 2015). The impact of bacteria on fruiting body formation on other model species of edible fungi such as the Basidiomycota *Agaricus bisporus* has been associated with remobilization or depletion of nutrients (Hayes et al., 1969; Masaphy et al., 1987; Kertesz and Thai, 2018). However, this interaction is more complex, as for instance, bacterial communities underneath fruiting bodies of multiple fungal species in natural forests are not only affected by the presence of the fruiting body, but also by environmental factors such as soil pH (Pent et al., 2017). A recent study investigating the microbiota in soils under ascospores of *Morchella sextelata* cultivated in a greenhouse showed that the bacterial communities in soils influenced by ascocarps differ significantly from those of the surrounding soils (Benucci et al., 2019). In addition, a second study following the progression of bacterial communities in natural soils during fruiting body formation in *Morchella rufobrunnea* showed that changes in the dominance of different functional groups in bacteria were correlated with different developmental stages of the fruiting body (Orlofsky et al., 2021).

Although there is a growing interest in understanding the relationship between *Morchella* and their associated bacteria, a systematic study analyzing bacterial communities associated with differentiated structures produced during the life cycle of *Morchella* is not yet available. In this study, we examined the composition of bacterial communities present in differentiated vegetative and sexual structures (mycelium and fruiting bodies) derived from diverse morel specimens collected in the wild, as well as mycelial cultures obtained from *Morchella* species in our laboratory culture collection. In addition, when possible, sclerotia, which are mycelial-derived survival structures that are proposed to be involved in primordia formation (Xu et al., 2022), were also sampled. Considering the distinct chemistry and function of these structures, we hypothesized that their associated bacterial communities should differ. We also hypothesized the existence of differences in the associated bacteriome between distinct phylogenetic clades (i.e., Elata versus Esculenta). Further, we isolated *Pseudomonas* spp. from these fungal isolates and characterized interactions between host and non-host *Morchella* species in co-culture assays, and used comparative genomics to identify unique sets of genes that may be involved in different types of interactions with the fungal hosts. This study offers the first evidence connecting observations from soils and confrontation studies indicating the relevance of *Pseudomonas* spp. on the physiology of morels.

## 2 Materials and methods

### 2.1 Collection and processing of the fruiting bodies

During the spring, from March to June 2019 and 2020, morel ascocarps were obtained from the canton of Neuchâtel. The fruiting bodies were initially identified at the clade level based on their morphology and later using molecular analysis based on the internal transcribed spacer (ITS) rDNA sequences (see below). The only ascocarp collected outside Neuchatel, was collected in 2020 (M20-7) in canton Vaud, Switzerland, and it was solely used for mycelial cultivation and the confrontation experiments with bacteria (see below). The *Verpa* spp. included in the community analysis were not genotyped but morphologically identified at the genus level. They were collected in the Canton of Neuchâtel in March 2019.

### 2.2 Culturing and isolation of mycelia

Upon collection of the ascocarps, several analyses were performed. First, material for genetic identification was collected directly upon sterilization of the tissue surface. Second, in those individuals with an intact fruiting body (16 individuals), fungal isolation was attempted either by germinating spores or by direct culturing pieces of hymenia. Isolation was performed on potato dextrose agar (PDA, Potato infusion powder, Sigma-Aldrich, 4 g/L + D(+) - glucose monohydrate, Roth, 20 g/L + Agar-Agar, Merck, 15 g/L). Pure isolates were obtained by successive plating in the same medium. In addition, two Chinese cultivars (NEU142 and NEU143) and one morel specimen from our collection (M84) were grown in PDA to be included in the analysis. Sclerotia were obtained by culturing on malt agar (Malt extract, Fluka, 12 g/L + Agar-Agar, Merck, 15 g/L; MA). All media were autoclaved at 121°C before pouring into Petri dishes.

### 2.3 DNA extraction and fungal identification

DNA was extracted either directly from fruiting bodies by cutting a piece of the hymenia, from mycelia by scraping hyphae from the surface of seven-day cultures grown on PDA, or from sclerotia sampled on mycelial cultures grown on MA. All DNA extractions were made with the Quick-DNA Fungal/Bacterial Miniprep Kit (ZymoResearch®) following the manufacturers protocol. After extraction, eluted DNA was quantified with a Qubit 2.0 with the Broad Range buffer and reagents (Invitrogen, USA). DNA was then diluted with PCR-grade water to a concentration of 2 ng/μL. Clades and potential species were assigned by sequencing the ITS region amplified with the primers ITS1F (5’-CTT GGT CAT TTA GAG GAA GTA A-3’) (White, 1990) and ITS4 (5’-TCC TCC GCT TAT TGA TA TGC-3’) (Gardes and Bruns, 1993). The PCR mix contained PCR-grade water, 2X ALLin™ Red Taq Mastermix and 0.2 µM forward and reverse primers, adding finally 1 µL of the DNA template concentrated at 2 ng/µL. The PCRs were performed in a Thermo Scientific Arktik thermal cycler with the following parameters: denaturation at 95°C for 1 min, 40 cycles of 95°C for 15 sec, 62°C for 15 sec, 72°C for 15 sec, and a final elongation at 72°C for 2 min, end at 20°C. Amplicons were purified in a MultiScreen® filter PCR µ96 (Millipore Corporation, USA) plate as follows: in each well, the PCR product and 50 μL of PCR-grade water was added; a vacuum of 20 bars was applied on the wells until those were dry; 20 μL of PCR-grade water was added to each well; after 2 min, DNA contained in the membrane from each well was resuspended by pipetting up and down 20 times. Once purified, PCR products were quantified by Qubit. The final concentration was adjusted at 2-40 ng/μL and sent to Genesupport (Geneva, Switzerland) for Sanger sequencing. After sequencing, the biological specimens of *Morchella* were attributed to the Elata or Esculenta *Morchella*’s clade using the ITS sequences (**Table S1**) and BLASTn (Altschul et al., 1990).

### 2.4 Sequencing of bacterial communities

The analysis of associated bacteria was made using the same fungal DNA extracts obtained above. The composition of associated bacteria was examined via amplicon sequencing of the V3-V4 region of the 16S rDNA. PCR amplification from the fungal DNA extracts followed methods described previously (Robinson et al., 2021). Briefly, a nested PCR approach was used to increase the amplification efficiency for bacteria with the relative exclusion of fungal mitochondria. The primers EUB9-27 5’-GAG TTT GAT CCT GGC TCA G-3’ and 907R 5’-CCG TCA ATT CCT TTG AGT TT-3’ (Weisburg et al., 1991) were used to amplify the V1-V5 region of the 16S rDNA. Purified PCR products from this initial amplification were sent to Genesupport (Geneva, Switzerland) for a nested amplification with primers to amplify the V3–V4 region (universal primers Bakt_341F 5’-CCT ACG GGN GGC WGC AG-3’ and Bakt_805R 5’-GAC TAC HVG GGT ATC TAA TCC-3’) (Herlemann et al., 2011). PCR amplification was performed along with sample barcoding to allow multiplexing and adapter ligation for sequencing on an Illumina MiSeq platform (2 x 300 bp paired end reads). No-template controls (starting with the DNA extraction) were also included in the sequencing run to identify and remove any contaminants.

### 2.5 Bioinformatic analysis of bacterial community data

Demultiplexed and trimmed sequences provided by Genesupport were analyzed using the QIIME2 pipeline (Bolyen et al., 2019). Read lengths were truncated to 488 bp to optimize total nucleotide lengths (based on quality scores). This allowed the joining of denoised paired-end reads by at least 12 identical bp. In order to obtain these sequences, the truncated, unmerged, paired-end reads were denoised using the DADA2 plugin (Callahan et al., 2016), which denoises paired-end sequences, dereplicates them, and filters chimeras. This step yields amplicon sequence variants (ASVs), which are expected to better represent the biological diversity present in the samples. As the DADA2 filtering step is based on quality score, there is no need for a quality filtering step prior to this. The ASVs were then taxonomically classified (**Table S2**) using QIIME2’s VSEARCH-based consensus taxonomy classifier (Rognes et al., 2016) with the SILVA database release 132 (Quast et al., 2013), which was previously trimmed to the same V3-V4 region produced by the sequencing primers used. Further data analysis was performed using the phyloseq (McMurdie and Holmes, 2013) package (version 1.30.0) and in R (version 3.6.2). First, any ASV represented in the no-template control samples were excluded. Any ASVs not assigned to the Bacteria Kingdom were discarded. The resulting dataset was normalized using a Total-sum scaling (TSS) implemented by the metagMisc package (version 0.0.4; github.com/vmikk/metagMisc). At this step, non-relevant genus rank taxonomies were merged with unassigned ones as “unassigned” ASV to be plotted against the “assigned” ASVs. Finally, samples were grouped by genus and ordered by sample types to be plotted as cumulative barplots.

### 2.6 Core community analysis

Data of ASV relative abundance for the three sample types (mycelium, sclerotia, and fructification) were used to define the core bacteriome. Given that the core community can be defined based on multiple parameters, two parameters were considered here: i) prevalence or occupancy (percentage of samples in which a given ASV is present), and ii) relative abundance threshold above which an ASV is considered as present in a given sample. The core microbiome was computed with the microbiome package (version 1.8.0) in R (Lahti et al., 2017). As there is no consensus on the prevalence and relative abundance thresholds that define the core community, we used an approach that defines consecutive values of interest for both parameters. Consequently, ASV prevalence values were set from 30% to 100% with steps of 5%. Detection thresholds for the ASV relative abundance were set to 0.05, 0.10, 0.15, 0.20, 0.30, 0.40, 0.50, 0.75, 1.00, 2.00, 5.00, and 10.00%. For each combination of prevalence and relative abundance threshold, a core community was calculated. Thereafter, based on the increasing number of ASVs included as core members, a Venn diagram was produced using R package nVennR (Perez-Silva et al., 2018) (version 0.2.3) for each sample type.

### 2.7 Bacterial isolation and characterization

Six bacterial strains were obtained during cultivation of the fungal mycelia. One of the strains was isolated during re-plating of the mycelium of from *Morchella* sp. M84 (B84). Two bacterial isolates were obtained from M19-34, one from a confrontation assay with another *Morchella* isolate (strain B33.4) and one when the fungus was grown alone (B188). Three strains (strains VD-NE ext, VD-NE ins, VD-NE white) were all obtained from mycelium of *Morchella* sp. M20-7, which was an ascocarp collected in 2020. Among those, the bacterial strain VD-NE ext was associated with the fungus after cultivation on a medium containing 100 µg/ml streptomycin. All the strains were initially identified by partial sequencing of the 16S rDNA amplified using the GMF3 (5’-AGA GTT TGA TC(AC) TGG C-3’) and GM4R (5’-TAC CTT GTT ACG ACT T-3’) primers (Muyzer et al., 1995).

### 2.8 Bacterial-fungal confrontations

Confrontations between *Pseudomonas* spp. and *Morchella* spp. were performed in 60 mm Petri dishes in PDA. Inocula for the fungal specimens were prepared on MA. Inocula for the bacterial strains were prepared from overnight liquid cultures grown in nutrient broth (NB; Roth) on a rotary shaker (120 rpm at room temperature (22-25°C). For the confrontation, a disc of agar from the edge of the fungal colony was cut and placed in the center of the Petri dish. To inoculate standardized concentrations of bacteria, overnight cultures were centrifuged and cell pellets were resuspended in Phosphate-buffered saline buffer (PBS; 137 mM NaCl, 2.7 mM KCl, 8 mM Na_2_HPO_4_, and 2 mM KH_2_PO_4_). An aliquot was diluted 100X and 5 µl were transferred to a Neubauer chamber for microscopic counting at 400X magnification. All strains were diluted to obtain an equal number of bacteria cells (77,000 cells/μl) to be inoculated in the confrontations. Inoculation was performed using a glass 10 µl pipette. A 5 µl drop of bacterial culture was inoculated at the periphery of the Petri dish. Pictures of the confrontations were taken at different days post inoculation. After approximately 22 days, confrontations were stored at 4°C until further use. Different variables were measured in the confrontations. All confrontations were performed in triplicate. Sclerotia formed during confrontation assays were transferred onto fresh MA plates to observe whether bacteria were associated with them. Furthermore, samples from the initial bacterial inoculation sites were taken and replated on nutrient agar (NA; NB supplemented with 1.5% technical agar, Biolife) with the fungicide cycloheximide (#C7698-5G from Sigma-Aldrich) to observe if bacteria survived the interaction. The same was done by inoculating a sample from the opposite site of the initial bacterial inoculation site.

### 2.9 Genomic DNA extraction, sequencing, and genome assembly

Bacterial genomic DNA was extracted using either QIAGEN® Genomic-tip 20/G (#10223 QIAGEN®) or the Wizard® HMW DNA Extraction Kit (#A2920 Promega, Switzerland). Protocols from QIAGEN® or Promega® were used and adapted to obtain sufficient quantities of high molecular weight genomic DNA. All bacteria were grown overnight in NB for genomic DNA extraction. The following modifications from the protocol provided by Promega® were applied: two additional cleanup steps with 1 ml PBS were done to remove any exopolysaccharide in the pellet; 20 μl of RNAse A (10 mg/ml, Roche catalogue number 10154105103) was added to each sample and incubated at 37°C for 30 min; Proteinase K (Sigma-Aldriech catalogue number 254-457-8) was added and incubated for 30 min at 56°C; after protein precipitation the sample was centrifuged at 16,000 x *g* for 20 min at 4°C; 0.7 volume of isopropanol was added to the volume extracted after the protein precipitation step; after that, the pellet was washed with ethanol, centrifugation was done at 4°C for 5 min at 16,000 x *g*; finally, 50 μl of rehydration solution was added. DNA quality was evaluated on a 0.7% agarose gel (tris-acetate-EDTA running buffer) containing 3 μl StainIN ™ GREEN Nucleic acid stain (highQu catalogue number NAS0201). The gels were run for 100 min at 70 V. If high molecular weight (HMW) DNA was present in the gel, quality measurements (ratios A260/A280 and A260/A230) were performed using a spectrophotometer (#ND-3300 Thermo Scientific ™). DNA quantification was measured using a fluorometer (Qubit 2.0 Invitrogen, USA). Pure genomic DNA samples with sufficient concentrations were sequenced with PacBio® at the sequencing facility of the University of Lausanne, Switzerland. The HMW DNA was sheared with Megaruptor (Diagenode, Denville, NJ, USA) to obtain 10-15 kb fragments. After shearing, the DNA size distribution was checked on a Fragment Analyzer (Advanced Analytical Technologies, Ames, IA, USA). Then, 500 ng of the DNA was used to prepare a SMRTbell library with the PacBio SMRTbell Express Template Prep Kit 2.0 (Pacific Biosciences, Menlo Park, CA, USA) according to the manufacturer’s instructions. The resulting library was pooled with other libraries processed in the same fashion. The pool was size-selected with Ampure beads (PacBio) to eliminate fragments <3 kb. Libraries were sequenced with v2.0/v2.0 chemistry and diffusion loading on a PacBio Sequel II instrument (Pacific Biosciences, Menlo Park, CA, USA) at 900 min movie length, pre-extension time of 2 h using one SMRT cell 8M. Genome assembly was performed using the protocol Microbial Assembly in SMRT Link Version 10.1.

### 2.10 Bacterial phylogenetic analysis

The phylogenetic assessment of *Pseudomonas* ASVs from the different sample types (mycelium, sclerotia, and fructification) and the isolated *Pseudomonas* strains (B188, B84, 33.4, VD-NE ext, VD-NE ins, VD-NE white) was conducted using as reference *Pseudomonas asiatica* strain RYU5 (NCBI accession number: NZ_BLJF01000001), *Pseudomonas helmanticensis* strain BIGb0525 Ga0304788_101 (NZ_SOCQ01000001), *Pseudomonas rhizosphaerae* strain DSM 16299 (NZ_CP009533), *Cellvibrio japonicus* strain ADPT1-KOJIBIOSE (NZ_CP043306), *Pseudomonas putida* strain KT2440 (LT799039), and *Pseudomonas carnis* strain MF6752 MF6752_contig_40 (NZ_JAENSU010000040). The 16S rDNA sequences were extracted from whole genomes using barrnap version 0.9 (Seemann, 2013) and imported into MEGA 11 (Tamura et al., 2021) along with the *Pseudomonas* ASVs for multisequence alignment using MUSCLE (Edgar, 2004). The best maximum likelihood model was determined out of 24 different nucleotide substitution models. The Kimura 2-parameter with Gamma distribution model was determined as the best fit. Following this step, a maximum likelihood tree was computed using this model with a bootstrap parameter set to 100 iterations. Percent identity matrix was computed based on MUSCLE alignment using R’s bio3d package (Grant et al., 2006).

### 2.11 Comparative genomic analysis

In order to perform genome comparisons, the six assembled *Pseudomonas* genomes were imported into an anvi’o (version 7) environment (Eren et al., 2021). Following the outlined pangenomic workflow, a contig database was populated using all the genomes in storage. DIAMOND (Buchfink et al., 2015) was used to calculate similarity at the protein level across all genomes. This step allowed us to define gene clusters based on similarities. Weak similarities were filtered out using minbit scores (default to 0.5) (Benedict et al., 2014). Then, cluster granularity in amino acid sequence similarity was determined using the Markov Cluster algorithm (van Dongen and Abreu-Goodger, 2012) with default parameters. The anvi-run-kegg-kofams program and then anvi-estimate-metabolism program were run to investigate the predicted metabolic capabilities. Briefly, the anvi-run-kegg-kofams program uses the KEGG database to annotate functions and metabolic pathways with hidden Markov model hits from KOfam, a database of KEGG Orthologs (KOs). Metabolic KEGG module completeness was evaluated with the anvi-estimate-metabolism program with a default threshold of 0.75 (i.e., 75% of the steps required in the KEGG module are present in a genome). The level of completeness for a given KEGG module (Kanehisa et al., 2014; Kanehisa et al., 2017) in our genomes (based on the previous KEGG annotation) was then assessed. Several modifications were done to organize gene clusters and genomes in the anvi’o interactive interface. At this point, manual binning selections were performed in order to highlight meaningful gene cluster groups. These bins were then exported to assess if metabolic capabilities were exclusive to an individual or to a group of genomes, and the level of completeness. In order to characterize the putative insecticidal toxin, we used the compare region viewer tool available at the Bacterial and Viral Bioinformatics Resource Center’s website. Basically, a feature (i.e., sequence) is blasted and the best hits genome regions are mapped in parallel. The direct access to the amino acid sequences allows further blasting against the NCBI’s non-redundant protein sequence database in order to clarify hypothetical proteins or unclear annotations. When necessary, protein sequences were submitted to the Motif search service on GenomeNet which queries sequences against the Pfam library (Bateman et al., 2002). The comparisons of the region were then displayed using the gggenes R package version 0.5.0 (https://CRAN.R-project.org/package=gggenes). In order to test for potential differences in CAZy gene counts, a reference database was built using cazy_webscraper (Hobbs et al., 2023). The uniprot database was interrogated for specific CAZy references for the *Pseudomonas* genus.

### 2.12 Bacterial tagging and co-cultivation of GFP-*Pseudomonas* with *Morchella*

Bacterial strains B33.4 and VD-NE white were tagged with the Green Fluorescent Protein (GFP) by electroporation, using the plasmid pUX-BF13 and the transposon Mini-Tn7 GFP Kanamycin-resistant. The tagged bacteria were maintained in 20% glycerol at -80°C. Overnight cultures on Luria Broth (LB, 10 g/L NaCl + 10 g/L Tryptone + 5 g/L Yeast extract, Sigma-Aldrich) at 18°C were made to get fresh cells for the experiments. Both tagged strains were co-cultured with *Morchella* sp. M19-43 in a drop-system, which consists of inoculating small amounts of bacterial and fungal cells in drops of liquid medium (Malt Broth, 12 g/L, Sigma-Aldrich) deposited in cell culture plates (Buffi et al., 2023). For this, a piece of growing mycelium (1 mm x 1 mm) was deposited in a 500 µL drop to which bacteria were added after 1 day (10 µL of 0.9% NaCl with 10^6^ cells/mL). The system was incubated at 22-23°C in darkness for seven days before being observed with an EVOSM5000 (Invitrogen, USA) inverted light microscope (bright field and green fluorescence). In addition, the interactions between tagged *Pseudomonas* strain VD-NE white and *Morchella* sp. M19-43 were observed with a SP8 Confocal microscope (Leica, Germany) by immersion of mycelial fragments from the co-culture in deionized H_2_O.

## 3 Results

### 3.1 Bacterial communities associated with a diverse *Morchella* population

**Figure 1:**
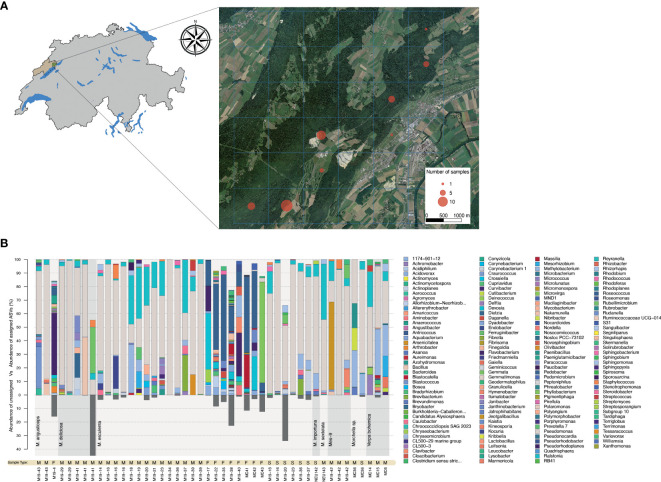
Identification of the morel bacteriome. **(A)** Sampling areas where wild morels were collected in 2019 in Neuchâtel, Switzerland. The number of fruiting bodies sampled is indicated by the circle size. **(B)** Bar charts displaying genus-level bacterial composition (relative abundance) with respect to the fungal source. The transition between different clades (bottom part of chart) is highlighted by a change in the grey background along the x-axis. Samples originating from mycelium (M), fruiting bodies (F), and sclerotia (S) are indicate as “Sample Types” below the bar plots. Three mycelium specimens from *Verpa* were included in this analysis, but excluded from the analysis performed afterwards. The proportion of unassigned ASVs is shown at the bottom of the graph by dark grey bars.

Bacterial community analysis was performed on a total of 28 biological specimens, including 25 that were collected in Spring 2019 in the canton of Neuchâtel (**Figure 1A**). The remaining specimens originated from our laboratory culture collection and included a wild morel (M84) and two strains isolated from soils in which spawn with a Chinese origin was previously used (*M. importuna* NEU142 and *M. sextelata* NEU143). The community analysis was performed on 10 intact fruiting bodies, 26 mycelial cultures, and 8 sclerotia, for a total of 44 samples (**Table S3**). The characterization of the bacteriome was carried out using sequencing of 16S rDNA amplicons obtained from fungal DNA extracts from vegetative and sexual structures. The bacterial communities associated with fruiting bodies were highly diverse, even among representatives from the Esculenta clade for which several samples were obtained (**Figure 1B**). In contrast, *Pseudomonas* was clearly the most abundant bacterial genus in the communities associated with mycelium and sclerotia in the vast majority of the samples (**Figure 1B**).

**Figure 2:**
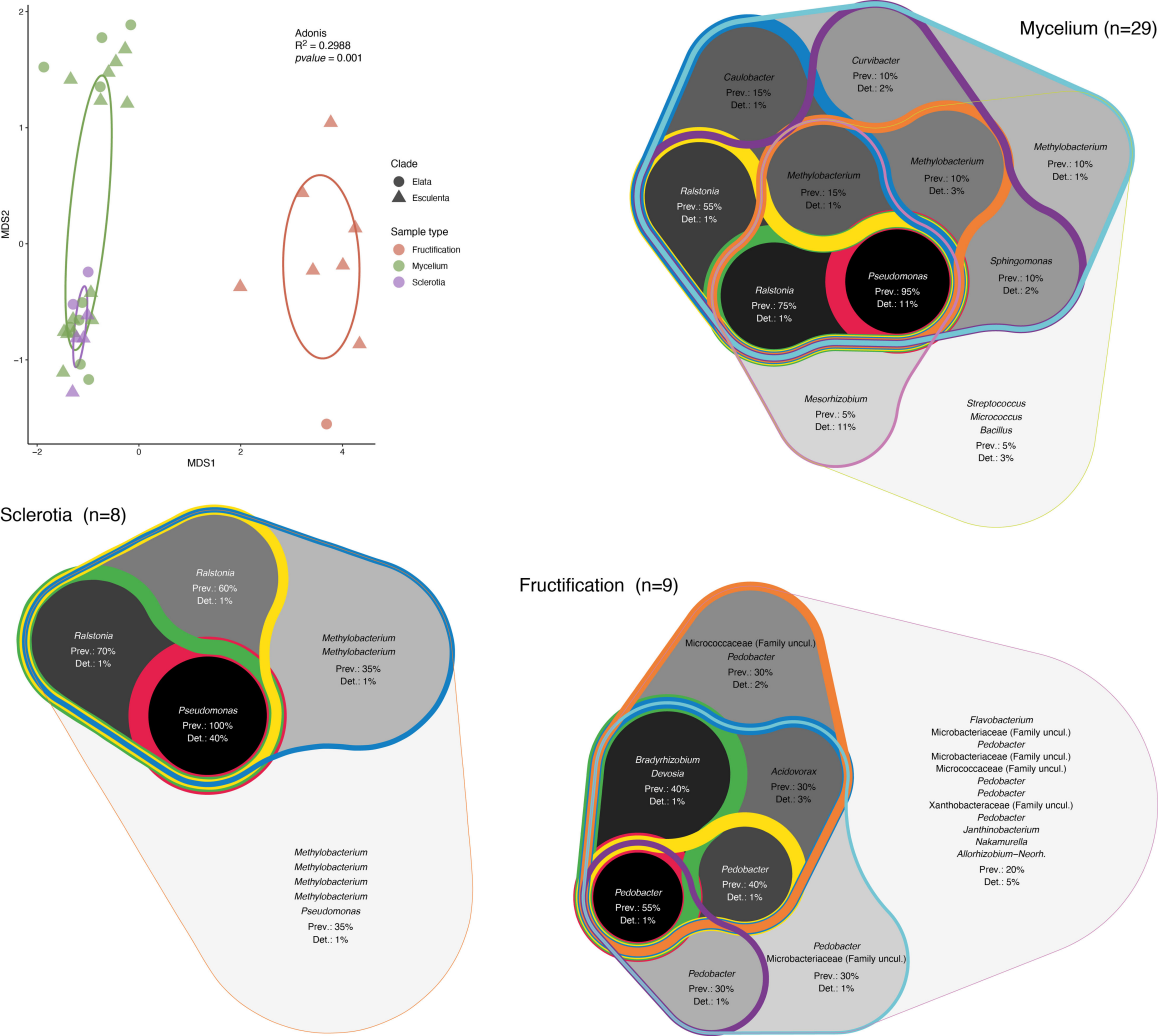
Analysis of the structure and composition of the morel bacteriome. Nonmetric multidimensional scaling (NMDS) analysis of the associated bacterial communities and core bacterial communities. Ellipses representing the 95% confindence in the NMDS plots are shown. The validity of different groups was assessed using Adonis and the results are also included in the plot. The core community was determined by combining ASV prevalence (Prev) and detection (Det) threshold (based on relative abundance). Different thresholds were applied to these parameters and the resulting core community was represented as a Venn diagram that encompasses the different thresholds of prevalence and detection of ASVs, with the highest values highlighted in a darker background and thicker lines (inner core) and the lowest values shown by lighter backgrounds and thinner lines (outer core).

In order to determine the effect of the morel clade on the structure of the associated bacterial communities, a nonmetric multidimensional scaling (NMDS) analysis was performed (**Figure 2**). In this analysis, the first dimension of separation distinguished communities from fruiting bodies from those in mycelia and sclerotia. In the case of fruiting bodies, it is difficult to assess if the clade played any role in community structure, as we only had a limited number of samples belonging to the Elata clade. However, for mycelium and sclerotia, the samples formed two groups along the second dimension, but those included samples intermixed from clades, indicating that a differentiation of the samples based on the *Morchella*’s clade is not supported by the results. In order to better compare the communities of the different vegetative and sexual structures investigated here, the core bacterial community for each structure type was established. The core bacterial community was defined based on the combination of prevalence (occupancy) and the threshold of detection (relative abundance) of a given ASV. As different arbitrary thresholds can be selected for each of these parameters, the core community was represented by integrating multiple prevalence and detection thresholds in the form of an inclusive Venn diagram. This graphical representation highlights the existence of multiple core layers, with the inner layers corresponding to the most ubiquitous taxa (highest prevalence and detection thresholds), while the outer layers correspond to increasingly marginal taxa (lowest prevalence and detection thresholds). This analysis indicated that *Pseudomonas* and *Ralstonia* correspond to the inner core bacterial community associated with mycelia (darker background in **Figure 2**), while *Methylobacterium* and *Ralstonia* were part of the outer core bacterial community (lighter background in **Figure 2**). The core bacterial community associated with sclerotia also indicated *Pseudomonas* as part of the innermost core, with ASVs related to *Ralstonia* and *Methylobacterium* composing the outer core community. In contrast, the core bacterial community from the fruiting bodies was more diverse and the prevalence of the ASVs in the innermost core was much lower (max. 55%) than the prevalence of the ASVs in the innermost core in both mycelia and sclerotia (95% and 100%, respectively). In the fruiting bodies, ASVs related to *Pedobacter* and *Bradyrhizobium* composed the innermost core community (**Figure 2**).

### 3.2 Isolation and identification of associated Pseudomonas spp.

**Figure 3:**
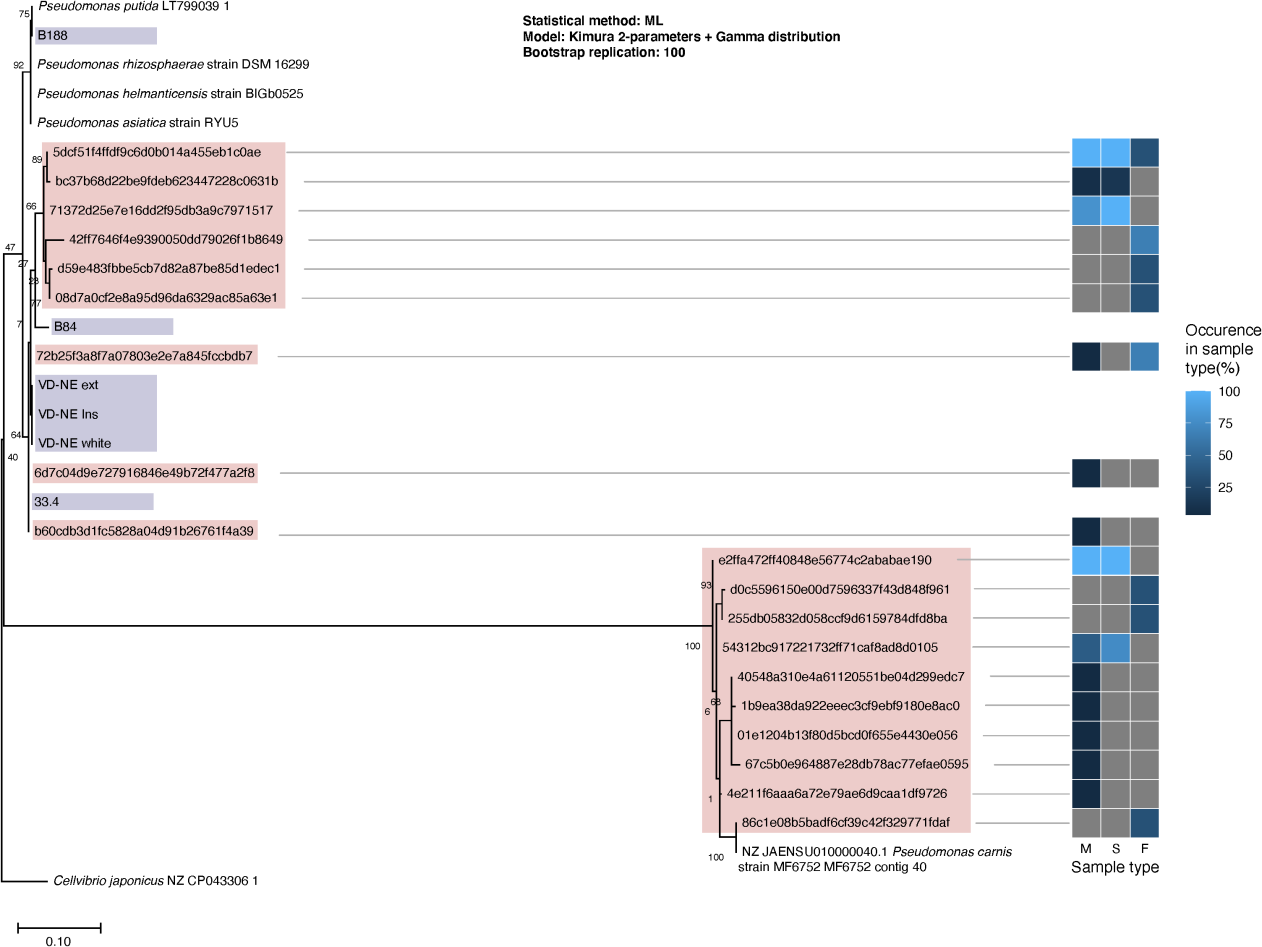
Phylogenetic classification of the *Pseudomonas* spp. isolated from *Morchella*. Maximum likelihood tree built from 16S rDNA sequences extracted from the reference genomes (branch labels with white background), whole sequenced genomes from the *Pseudomonas* spp. isolates (violet) and V3-V4 16S rDNA regions obtained from *Pseudomonas*’ ASVs (pink) from the fungal-associated bacterial communities. The heatmap to the right indicates the occurrence of the ASVs in the different source sample types: M, mycelium; S, sclerotia; F, fruiting bodies.

The detection of *Pseudomonas* as the most common bacterial genus associated with mycelium and sclerotia of *Morchella* is noteworthy as, in parallel to the molecular analysis, we isolated six bacterial strains classified as *Pseudomonas* spp. based on their 16S rDNA sequence. The bacteria were isolated during the cultivation of fungal mycelia, or were shed from apparently axenic mycelium in confrontation experiments between various fungal strains (**Supplementary Figure 1**; **Tables S4**, **S5**). A phylogenetic comparison based on the 16S rDNA of the bacterial isolates, reference *Pseudomonas* strains, and the ASVs assigned to *Pseudomonas* within the bacteriome, clearly showed that the B33.4, VD-NE ext, VD-NE ins, VD-NE white strains, were not only closely related among them, but also to ASVs found within the mycelial *Morchella*’s bacteriome (**Figure 3**). In contrast, strains B84 and B188 were more distantly related to strains belonging to the *Morchella*’s bacteriome. However, none of the isolated strains corresponded to ASVs that were part of the innermost core bacterial community. Instead, they were related to ASVs that were less prevalent or abundant. The closest cultured relative to the inner core *Pseudomonas* ASVs corresponded to the species *Pseudomonas carnis* strain MF6752 (**Figure 3**).

### 3.3 Confrontation assays between Pseudomonas spp. and Morchella spp.

**Figure 4:**
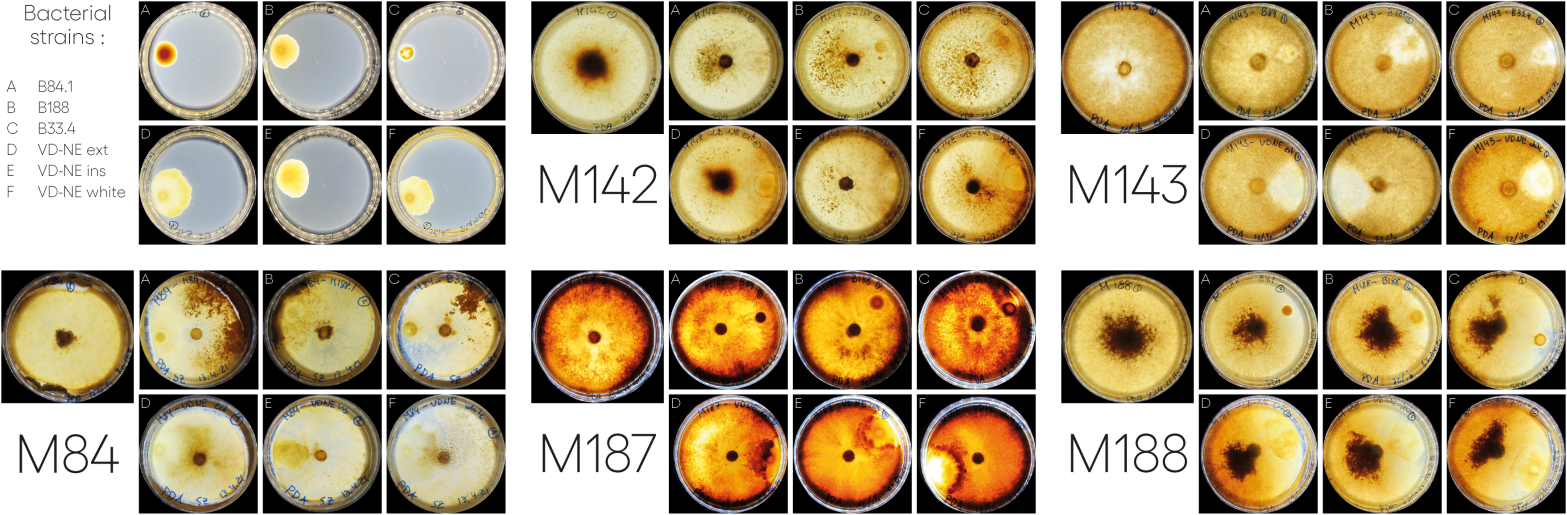
Interactions between different *Morchella* spp. and *Pseudomonas* spp. isolated from *Morchella* mycelia. In each case, the bacterium was inoculated on one side of the plate and the fungus was inoculated as a plug in the center. Images of the interactions taken at 21 or 22-days post inoculation. For the bacterial strains (top left): A= B84; B=B188; C=33.4; D=VD-NE Ext; E=VD-NE Ins; F= VD-NE White. For the *Morchella* specimens, the control plates are shown on the left for each panel. M142, *M. importuna*; M143, *M. sextelata*; M84, *M. rufobrunnea*; M187, *Morchella* sp. M19-29; M188, *Morchella* sp. M19-34.

We next tested the interaction of the six bacterial strains with five *Morchella* strains; we the included the fungal hosts from which the bacterial strain B84 (*Morchella rufobrunnea* M84, which is a strain grown for several decades in the laboratory) and the strains B188, and B33.4 (*Morchella* sp. M19-34) were derived from. In addition, we included an environmental representative of the Elata clade (*Morchella* sp. M19-29) and two strains from our culture collection that were isolated from European fields used for the cultivation of morels from spawn prepared in China (*M. importuna* M142 and *M. sextelata* M143). Confrontation assays were performed in paired growth assays on agar plates. The bacteria and fungi achieved growth in the medium, however the interactions observed between different bacterial-fungal pairings varied and resulted in distinct bacterial and fungal growth phenotypes (**Figure 4**).

There were two general growth patterns of the bacterial colonies (**Figure 4**). Strains B188, B84 and B33.4 developed as a round colony that rarely exceeded 1 cm in diameter. In contrast, all the VD-NE strains grew as spreading colonies. Fungal growth was differentially affected by the interaction. For *M. rufobrunnea* M84, a large number of sclerotia were induced by the interaction with strains B188, B84 and B33.4, but growth was restricted by the VD-NE strains. A large number of sclerotia were produced on the opposite side of the inoculation area upon the confrontation of the environmental *Morchella* sp. M19-34 with all the bacteria. This fungal strain, which was the host of the *Pseudomonas* strain B33.4, was partially inhibited by this bacterium and the related VD-NE strains. Growth of the three Elata strains tested (*M. importuna* M142, *M. sextelata* M143, and *Morchella* sp. M19-29) was less inhibited in the confrontations with all the bacterial strains. For *M. importuna* M142, sclerotia were also produced on the opposite side of the inoculum. In contrast, sclerotia were never produced by the environmental strain *Morchella* sp. M19-29 or *M. sextelata* M143. In these two fungal strains, a difference in the deposition of a dark pigment was observed in response to the interaction in particular with bacterial strain B33.4 and the three bacterial VD-NE strains. The area of contact with the bacterial inoculum usually remained un-pigmented for *M. sextelata* M143 when interacting with all the bacterial strains, with the sole exception of confrontations with B84. *Morchella* sp. M19-29 produced a dark line between itself and the bacterium (conflict line) in the interaction with strains associated with ASVs of the mycelium bacteriome (strains 33.4 and VD-NE). The bacteria always survived in the area of inoculation.

### 3.4 Comparative genomics of Morchella-associated Pseudomonas

In order to better understand the underlying genetic determinants and mechanisms that could explain the different interacting phenotypes, the genomes of the six *Pseudomonas* strains were sequenced and analyzed. The complete genomes were in the range of 6.1-6.6 Mbp and coded for approximately 5,700 to 6,000 predicted coding sequences (CDS). The genome of strain B188 contained the largest number of tRNAs, while strain B84 contained the lowest number. The bacterial strains that were closely related in the phylogenetic analysis (B33.4 and VD-NE strains) all have similar genome sizes and GC content, but strain B33.4 has a lower number of CDS and tRNA, and a low number of repeat regions. The number of hypothetical proteins and those with a functional assignment was very similar between the strains (**Table 1**). Also, the two strains originating from the same fungal host (B84 and B33.4) diverged in the GC content, repeat regions, tRNAs, and CDS. In addition, the annotated genomes were thoroughly scanned based on Uniport protein names, gene symbols, EC number, and CAZy family names. Five CAZy families were found in all the genomes. They corresponded to glycoside hydrolase, glycosyltransferase, polysaccharide lyase, carbohydrate esterase, and carbohydrate-binding module families. The number of hits was very similar overall and for the breakdown into the different families, and corresponded to 100, 96, 105, 102, 101, 102, respectively, in the genomes of the strains B33.4, B188, B84, VD-NE ext, VD-NE ins, and VD-NE white (**Supplementary Figure S2**).

**Table 1:** Genome statistics of *Pseudomonas* strains isolated from *Morchella* spp.

Genome	B188	B84	B33.4	VD-NE ins	VD-NE ext	VD-NE white
Contigs	1	1	1	1	1	1
GC Content	61.51	61.41	59.15	59.22	59.22	59.22
Genome Length (bp)	6’191’592	6’630’422	6’604’192	6’696’054	6’696’047	6’695’895
CDS	5701	6029	5917	6080	6081	6084
tRNA	114	66	73	89	89	91
Repeat Regions	76	57	44	73	73	73
rRNA	22	19	19	19	19	19
Hypothetical proteins	1’102	1’411	1’323	1’407	1’412	1’414
Proteins with functional assignments	4’599	4’618	4’594	4’673	4’669	4’670
Proteins with EC number assignments	1’240	1’281	1’262	1’259	1’258	1’258
Proteins with GO assignments	1’060	1’097	1’088	1’086	1’085	1’085
Proteins with Pathway assignments	926	969	956	949	948	948
Proteins with PATRIC cross-genus family (PGfam) assignments	5’654	4’933	5’669	5’904	5’903	5’907

**Figure 5:**
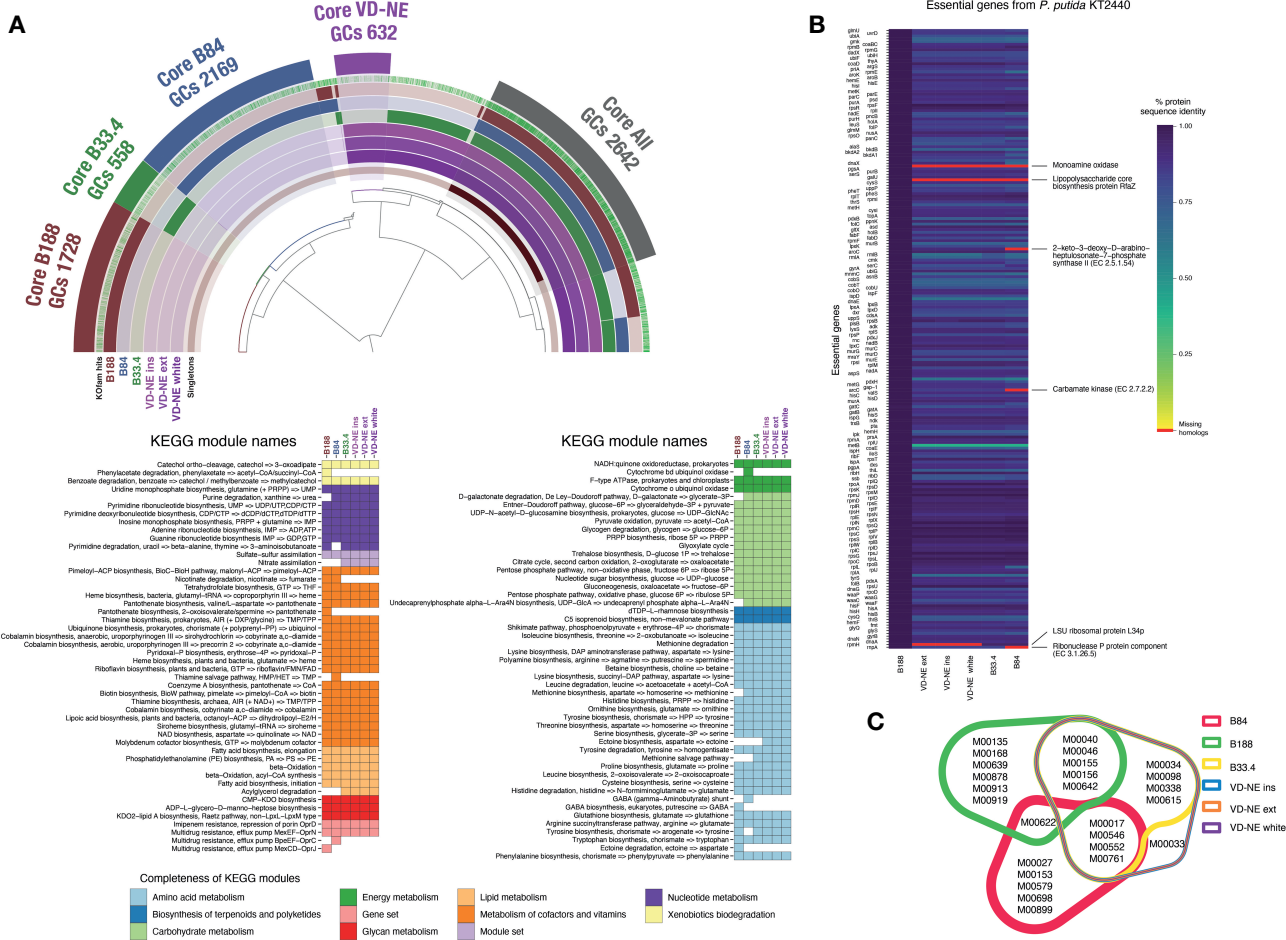
Comparative analysis of the genomes of the six *Pseudomonas* spp. strains isolated from *Morchella*. **(A)** Analysis of core and unique gene clusters (GCs) and KEGG modules shared between the strains. The number of GCs within the core genomes of each strain or group of strains (VD-NE strains) is indicated. The colored heatmaps below show the presence of complete KEGG modules in the genomes. **(B)** Comparison with the essential gene content of the model organism *P. putida* KT2440. The missing genes in the genomes of the *Morchella*-associated *Pseudomonas* are indicated in red. **(C)** Shared and unique KEGG modules found in the genome shown as a color-coded Venn diagram.

The shared part of the genome of the six strains corresponded to 2,642 gene clusters (GCs), which is approximately half of the genome with a functional assignment. Accordingly, a large fraction of the central metabolism modules (e.g., carbohydrate metabolism, nucleotide and amino acid metabolism) was shared between the strains. In contrast, the completeness of other metabolic modules was variable, including those involved in the metabolism of cofactors and vitamins (**Figure 5A**). The three VD-NE strains were very closely related. Not only did they share all of the annotated metabolic modules, but also had a common core of 632 GCs (**Figure 5A**). In contrast, the genomes of the three other strains (B188, B84, B33.4) all contained a set of unique specific GCs. This set was largest for strain B84 (2,169 GCs), and smallest for strain B33.4 (558 GCs), which was also closely related to the VD-NE strains.

We next compared the gene content of the strains using the essential gene content of the model organism *P. putida* KT2440 as a reference. The essential gene repertoire of KT2440 is not only fully present in strain B188, but also, the protein sequence identity between the genes is extremely high (**Figure 5B**). In contrast, the monoamine oxidase and the lipopolysaccharide biosynthesis protein RfaZ were missing in the other five strains. The LSU ribosomal protein L34p was missing in the VD-NE strains, while the 2-keto-3-deoxy-D-arabinoheptulosante-7-phophate synthase II, carbamate kinase, and the ribonuclease P protein components were missing in strain B84. Moreover, compared to B188, the protein sequence identity for all the other strains was much lower. The strains B188 and B84 contained the highest number of unique complete KEGG modules (6 and 5, respectively; **Figure 5C**). In the case of B188, two of these modules correspond to metabolic components of plant metabolism, including the GABA biosynthesis pathway (M00135) and the Crassulacean acid metabolism (M00168). In contrast, strain B84 contained a GABA shunt module (M00027). With the exception of the strain B84, all the other strains possessed the multidrug efflux pump MexJK-OprM module (M00642). In addition, strains B188 and B84 possessed the efflux pumps MexCD-OprJ (M00639) and BpeEF-OprC (M00698), respectively. The only complete KEGG module that distinguished the VD-NE strains from the other strains, was module M00033, which encodes the biosynthesis of the compatible solute ectoine from the amino acid aspartate.

**Figure 6:**
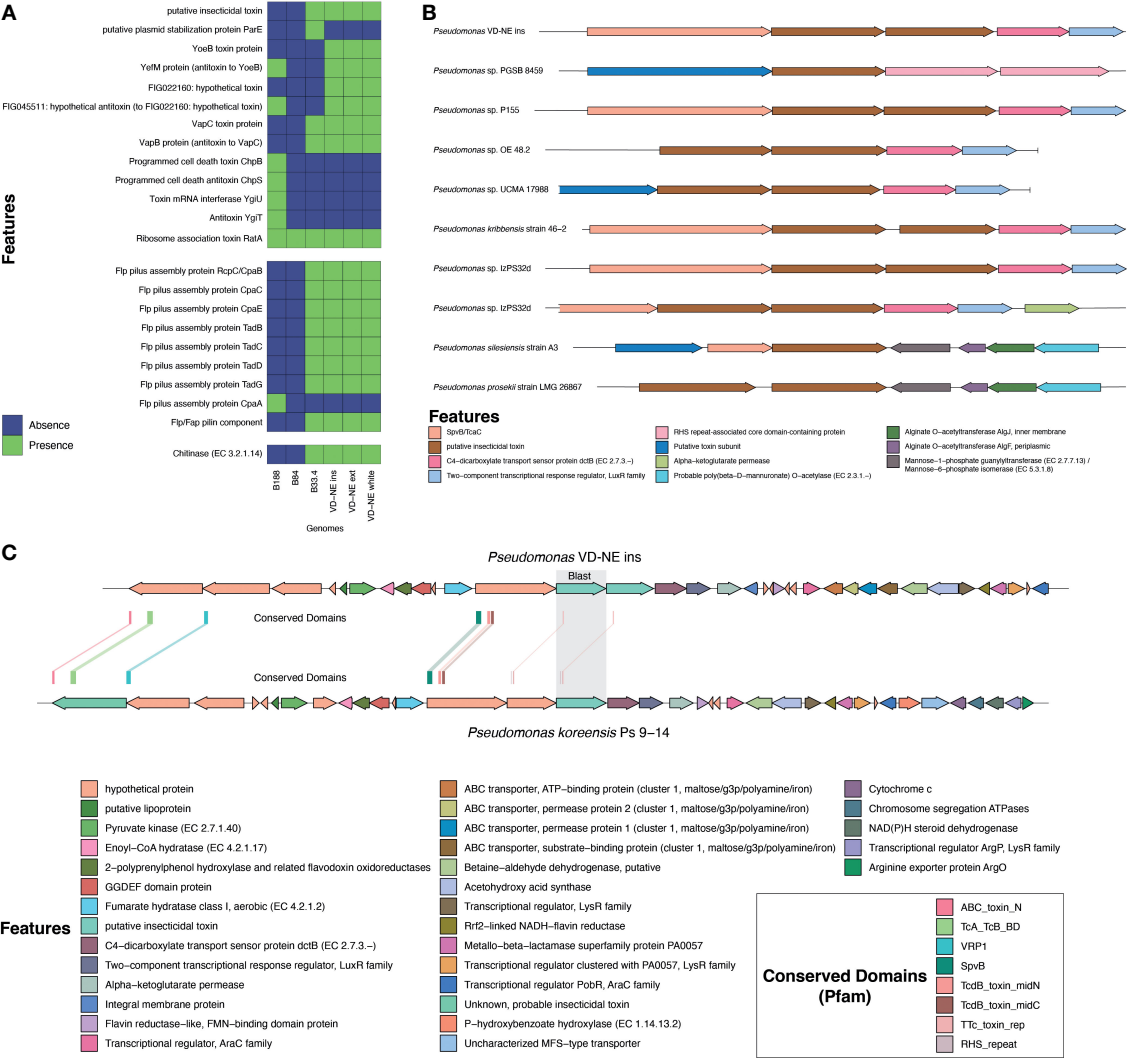
Analysis of genomic features associated with the interaction of bacteria with a eukaryotic host. **(A)** Presence and absence of toxins, appendages involved in cell adhesion, secretion systems, and chitinolytic or biocidal enzymes based on annotation. **(B)** Comparative analysis of the genomic region containing the putative insecticidal toxin. **(C)** In-depth analysis of the genome region containing the putative toxin cluster in comparison to *Pseudomonas koreensis* highlighting conserved domains found in the toxin-related genes.

Genomic features implicated in the interaction of bacteria with a fungal host such as toxins, appendages involved in cell adhesion, secretion systems, and chitinolytic or biocidal enzymes were differently distributed among the strains. All strains had a complete set of genes involved in flagellar synthesis (**Supplementary Figure 3**). The strains related to ASVs from the core bacteriome (VD-NE strains and strain B33.4) were characterized by the presence of an insecticidal toxin, as well as by the presence of adhesive fimbria and chitinases, which were absent from the B188 and B84 strains (**Figure 6A**). Moreover, a number of toxin-antitoxin systems, were also differentially distributed between the strains related to ASVs of the bacteriome, as compared to the B188 and B84 strains.

Insecticidal toxins are part of the features shared by root-colonizing fluorescent pseudomonads with the ability to suppress fungal pathogens, and therefore, it was surprising to find a putative insecticidal toxin as part of the shared genes for the strains closely related to ASVs in the *Morchella* bacteriome. However, these kinds of features might explain the phenotype observed in the interactions with these strains. Therefore, this insecticidal toxin was investigated in more detail. For this, the genomic context and the presence of similar toxins in other pseudomonads was evaluated. The genome of strain VD-NE Ins was used as reference for the other VD-NE strains. In this genome, two identical copies of the putative insecticidal toxin were present in tandem (**Figure 6B**). The same configuration was found in seven additional pseudomonads. A protein containing a SpvB/TcaC N-terminal domain and a C4-dicarboxylate transport sensor protein flanked the two identical copies of the toxin. Domains constituting hallmarks of the toxin complex in other pseudomonads were identified in the genomes of the *Morchella*-associated strains (**Figure 6C**). Interestingly, these strains partly inhibited fungal growth in the interaction tests (**Figure 4**).

**Figure 7:**
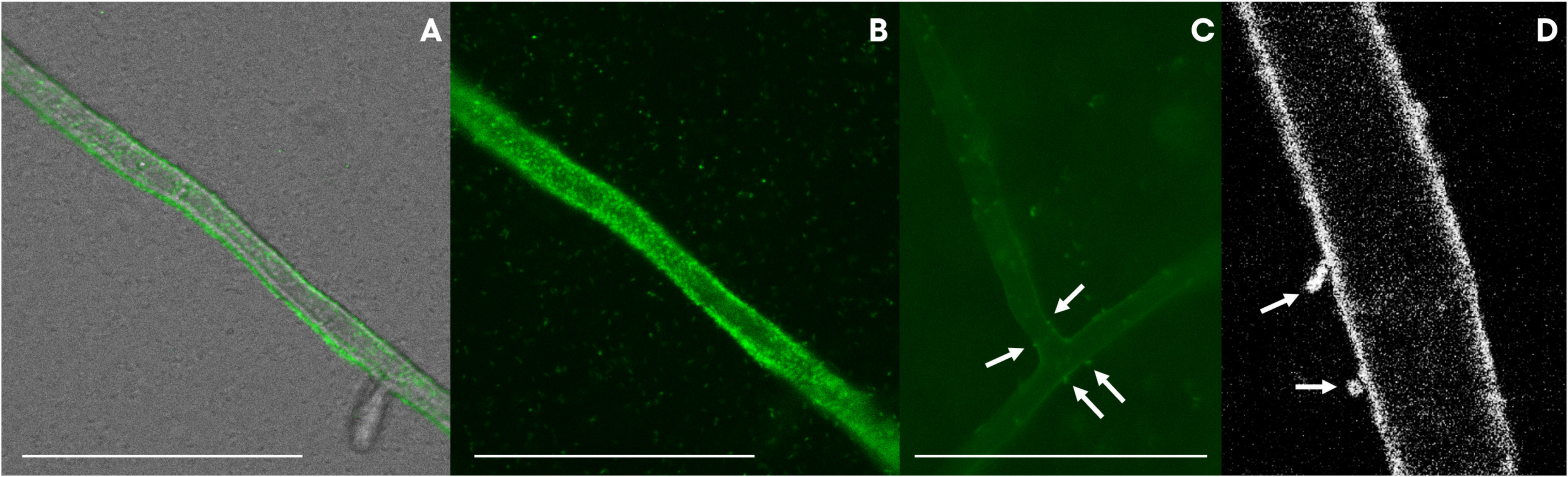
GFP-tagged *Pseudomonas* strains VD-NE white **(A, B, D)** and 33.4 **(C)** associated with hyphae of *Morchella* sp. M19-43. Pictures were taken by inverted light microscopy [**(A–C)**, magnification 400x, deionized water] and confocal microscopy [**(D)**, magnification 63x, deionized water].

The presence of a Flp pilus was also diagnostic of the strains related to ASVs in the bacteriome. Therefore, we performed additional experiments to validate the ability of the strains to attach to the fungal mycelium. Microscopic observations revealed that the bacteria not only used the fungal hyphae to move (i.e., as fungal highways), but also, were capable of attaching to the hyphae (**Figure 7**). In co-culture experiments performed with a GFP-tagged strain of the VD-NE White strain, the bacterium was present in higher amounts interacting with the fungal hyphae. In contrast, the cells of the strain B33.4 were mainly located at the bottom of the Petri dish, but the few cells associated with the fungus were also attached to the hyphae.

## 4 Discussion

In this study, the bacteriome of the fungal genus *Morchella* was investigated in detail for the first time. This fills an important knowledge gap in understanding the ecology of this fungal group; the relationship of *Morchella* spp. with their associated bacteria is of high interest, as bacteria are considered to be a factor promoting primordia differentiation and fruiting body growth, as well as acting in the control of diseases that could affect crop yield in cultured systems (Liu et al., 2017). A previous study investigating the bacterial communities in greenhouse soils cultivated with *M. sextelata* found that bacteria such as *Pedobacter*, *Pseudomonas*, *Stenotrophomonas*, and *Flavobacterium*, comprise the core microbiome in soils underneath fruiting bodies (Benucci et al., 2019). From those, only *Pedobacter* was found as a component of the inner core bacteriome of the fruiting body of the Swiss populations investigated here (**Figures 1**, **2**). Instead, other genera such as *Deviosa*, *Bradyrhizobium*, and *Acidovorax* constituted the core bacteriome of fruiting bodies. From those, the *Deviosa* and *Bradyrhizobium* genera have been shown to be dominant members of the mycosphere of the ectomycorrhizal Basidiomycota fungus *Cantharellus cibarius*. In *C. cibarius*, unsuccessful trials for its cultivation under controlled conditions have been performed. The failure to cultivate this species has been partly assigned to the difficulties in replicating the complex associations that it establishes with multiple bacterial species (Ge et al., 2023). Moreover, in the case of *Bradyrhizobium*, its dominance in fruiting bodies in truffles has been suggested to be relevant for the provision of additional nitrogen through symbiotic nitrogen fixation (Antony-Babu et al., 2014; Benucci and Bonito, 2016), which is also suggested to be the case for *Morchella* (Yu et al., 2022). More recently a study investigating the changes in the soil microbiome during the cultivation life cycle of *M. sextelata* has revealed a very dynamic impact of the fungus life cycle on the soil microbiome (Zhang et al., 2023). In this latest study, the composition of the communities differed significantly for three growth stages (i.e., mycelium, primordia, fruiting body). Most of the groups identified here in the core microbiota of *Morchella* were detected in the soil microbiome, but mainly in the stage of primordia formation.

Regarding the specific bacteriome of the vegetative structures studied here, namely mycelium and sclerotia, we observed that the bacteriome differed substantially from the bacteriome of fruiting bodies (**Figures 1**, **2**). The main difference corresponded to the high abundance of *Pseudomonas* in both mycelial and sclerotia samples. *Pseudomonas* spp. have been among the most commonly cited bacterial genera establishing inter-kingdom associations with fungi (Probst et al., 2023), including in the context of co-infections (Grainha et al., 2020; Santos-Fernandez et al., 2023). *Pseudomonas* spp. have remarkably varied lifestyles matched by the production of multiple secondary metabolites that can support diverse physiologies (e.g., iron scavenging, swarming motility, biofilm formation, induced cell death) (Tarkka et al., 2009). In the past, *Pseudomonas* in co-cultivation with *Morchella* have been shown to serve as a potential nutritional resource (Pion et al., 2013), or promote the exploitation of organic nitrogen by the fungus (Lohberger et al., 2019). This has prompted some authors to hypothesize that *Pseudomonas* spp. may have an effect on morel primordial differentiation, which is a key stage in the formation of fruiting bodies (Li et al., 2017). *Pseudomonas* was the main biomarker among the enriched members of the soil microbiota associated to the phase of mycelial growth. In this phase, pathways related to carbohydrate, amino acid and energy metabolism were enriched, supporting the role of the soil microbiota (and among those *Pseudomonas*) in nutrient absorption by the mycelium (Zhang et al., 2023). Our results provide a clear indication that *Pseudomonas* spp. are natural biological partners of diverse *Morchella* species, regardless of the clade to which they belong, and that their distribution changes according to the vegetative and reproductive structure under consideration, whereby they are significantly less represented in fruiting bodies compared to mycelium and sclerotia.

Comparative transcriptomic studies during early stages of fruiting body formation in *M. importuna* have shown that carbohydrate catabolism and energy metabolism are significantly enhanced in the young fruiting body stage as compared to the mycelial stage. In addition, genes for heat shock proteins also have a higher expression in the fruiting body stage (Ge et al., 2023). Another study comparing mycelial growth and sclerotia has shown that genes related to the catabolism of carbohydrates are upregulated mainly during the vegetative mycelium growth stage, while the anabolism of the energy-rich substances occurred in both mycelial growth and during sclerotial morphogenesis (Liu et al., 2019). Combined transcriptomic and metabolomic studies have confirmed a considerable shift during vegetative growth and sclerotial formation and maturation (Fan et al., 2023). These substantial changes clearly have an effect on the composition of the bacteriome, as the results obtained in our study confirmed our initial hypothesis regarding the divergence of the bacteriome from vegetative and sexual structures. However, concerning our initial hypothesis of the divergence of the bacteriome between individuals from different clades, the data for mycelia and sclerotia did not support such a differentiation.

We also show that some of the *Pseudomonas* that are part of the bacteriome can be isolated from the fungal host and cultured as pure isolates. Those strains might represent facultative associates, while the most intimately associated *Pseudomonas* (those constituting the innermost core bacteriome; **Figure 2**), could not be recovered by bacterial isolation (**Figure 3**). The inability to isolate bacteria related to ASVs from the innermost core bacteriome could be the result of a highly specialized metabolism, or dependence on the host’s metabolism. Based on the partial 16S rDNA sequence, the closest cultured reference strain to the core bacteriome ASVs corresponded to *P. carnis*, which is a relatively recently described *Pseudomonas* species involved in meat-spoilage (Lick et al., 2020). Cultured representatives of this species have never been shown to interact with fungi. In addition, the lack of resolution of the 16S rDNA as a molecular marker for the identification of closely related species, suggest that the *Morchella*-associated ASVs from the innermost core bacteriome might have a very unique metabolism that precludes cultivation in the absence of the fungal host.

Based on a phylogenetic analysis using the 16S rDNA, the isolated bacteria were classified as belonging to the subgroups *Pseudomonas koreensis* (strains B33.4, VD-NE ext, VD-NE ins, VD-NE white), *Pseudomonas baltica* (strain B84), and *Pseudomonas putida* (B188) (Girard et al., 2021). Different types of interactions have been observed *in vitro* between *Morchella* and *Pseudomonas* spp (Pion et al., 2013; Lohberger et al., 2019). The *Morchella-*associated bacterial strains related to ASVs from the bacteriome (strain B33.4 and the three VD-NE strains) elicited a stronger negative interacting phenotype in the five fungal strains tested, as compared to the two strains that were not related to ASVs from the bacteriome (strains B188 and B84, **Figure 4**). This could seem surprising, as it can be expected that bacterial associates that are clearly a part of the bacteriome might have a positive effect on the fungal host. However, a previous study in another bacterial-fungal partnership between the fungus *Mortierella elongata* AG77 and *Mycoavidus cysteinexigens*, its bacterial endosymbiont, has shown that the fungus, when cured from the endosymbiont, displays higher growth rates suggesting a fitness cost on the host to harbor endosymbionts under the conditions tested (Uehling et al., 2017). A mechanism of incompatibility between related bacteria, to avoid a fitness cost of bacterial competition, might explain the results in our experiment. Moreover, all of the bacteria triggered sclerotia formation in the three fungal strains, resembling the phenotype observed in the farming of bacterial cells described in the interaction between *M. esculenta* and *P. putida* (Pion et al., 2013). This phenotype was triggered by the use of the bacterium as a nutrient source, something that could be investigated in the future with the strains identified in this study. Sclerotia formation has been considered as a key stage in fruiting body formation (Ower, 1982), and the effect of the associated bacteria on this process could contribute to improving morel cultivation conditions in the future.

The fact that all the bacterial strains isolated here possessed relatively large genomes and encoded the vast majority of essential genes of other model free-living *Pseudomonas* spp. hints at a facultative association to the fungal host, in comparison to the functions identified so far within the genomes of other stricter fungal endosymbionts (Guo et al., 2020). Moreover, differences in the genomes of the bacterial strains were consistent with the differential interacting phenotypes in the confrontation assays. The comparative genomic analysis of the strains showed that those that were part of the core bacteriome (i.e., B33.4, VD-NE ext, VD-NE ins, VD-NE white) and those that could not be directly related to ASVs in the fungal bacteriome (i.e., B188 and B84) have very distinct genomic repertoires (**Figure 5**). More specifically, genes previously known to be involved in interactions of bacteria with fungal hosts such as toxins, appendages involved in cell adhesion, and chitinolytic or biocidal enzymes, were only found in the strains related to ASVs from the core bacteriome (strain B33.4 and the three VD-NE strains) (**Figure 6**).

The production of an insecticidal toxin (*fluorescens* insecticidal toxin or Fit) has been suggested as allowing the use of insects as a secondary ecological niche for root-colonizing fluorescent pseudomonads (Ruffner et al., 2015; Flury et al., 2016; Arrebola et al., 2022). In the case of the *Morchella*-associated *Pseudomonas*, the putative insecticidal toxin detected does not bare resemblance to Fit. Rather, a genomic region with homology to toxin clusters was detected (**Figure 6**). Toxin clusters with insecticidal activity were first identified in the entomopathogen *Photorhabdus luminescens* (Blackburn et al., 1998; Bowen et al., 1998; ffrench-Constant and Bowen, 2000). Other clusters with homology to those of *P. luminescens* have been found to contribute to toxicity in other bacteria such as *Yersinia enterocolitica* (Tennant et al., 2005). The toxin clusters from *P. luminescens* are composed of three subunits: TcA, TcB, and TcC, with the first one acting as an injecting device responsible for translocating the actual toxic component into host cells (Leidreiter et al., 2019). Likewise, toxin clusters have been identified in *Pseudomonas taiwanensis*, which is a species from soils with a broad-host range of insecticidal activity against a Dipteran and Lepidopteran species (Chen et al., 2014). Similarly, some strains in the *P. fluorescens* group have genes similar to those encoding toxin complex proteins (Rangel et al., 2016). These types of toxins are being studied for their potential as novel compounds that could be relevant in biocontrol (Leidreiter et al., 2019). In addition, mutational studies have indicated that chitinase C and phospholipase C are essential for the insecticidal activity (Flury et al., 2016). Neither insecticidal toxins nor a chitinase were detected in the strains B188 and B84, but were consistently detected in the genome of the ASVs from the core bacteriome (B33.4 and the three VD-NE strains). Therefore, in the future, it would be interesting to evaluate their role in regulating the interaction of *Morchella* with other organisms such as insects or fungivorous nematodes.

Another striking identifying feature of the strains closely related to ASVs from the bacteriome is the putative presence of a Flp pilus, which have been detected previously on pathogens of eukaryotic hosts, but also on the plant growth promoting and mycorrhizal enhancer *P. fluorescens* C7R12 (Bergeau et al., 2019). In this bacterium, the pili are implicated in the adhesion to environmental surfaces. This ability was demonstrated experimentally in the strains B33.4 and VD-NE White, as the bacteria were able to attach and move on the hyphae of *Morchella* (**Figure 7**).

A large number of type II toxin-antitoxin (TA) systems were also detected in the bacterial isolate genomes and distinguished the different strain groups. Type II TA systems are small genetic elements composed of a toxin protein and its cognate antitoxin protein, which would counteract the toxicity of the toxin component. Multiple functions have been proposed for the Type II TA systems including a stress response to starvation or the emergence of persister cells (Fraikin et al., 2020). In addition, a study on the endobacterium *Candidatus* Glomeribacter gigasporarum showed that the gene expression of two TA systems (YoeB/YefM and ChpB/ChpS) changed during the life cycle of the fungal host. This study suggests that the TA system helps the endobacterium to adapt to its intracellular habitat (Salvioli di Fossalunga et al., 2017). The same TA system, YoeB/YefM, which was present in the three VD-NE strains, might play a similar role in their putative endohyphal lifestyle.

In summary, in this study we have shown that a long-suspected interaction between *Morchella* and *Pseudomonas* is supported by the detection and isolation of bacteria from this genus from mycelium and sclerotia. Our study also demonstrates a drastic change in the associated bacteria upon fruiting body formation that warrants further investigation to improve our understanding of the complex life cycle of these emblematic fungi.

## Data availability statement

The datasets presented in this study can be found in online repositories. The names of the repository/repositories and accession number(s) can be found in the article/**Supplementary Material**.

## Author contributions

GC: Data curation, Formal analysis, Investigation, Methodology, Visualization, Writing – original draft, Writing – review & editing. BH: Data curation, Formal analysis, Investigation, Writing – review & editing. MC: Data curation, Formal analysis, Investigation, Methodology, Visualization, Writing – original draft, Writing – review & editing. SZ: Data curation, Formal analysis, Investigation, Methodology, Visualization, Writing – review & editing. PH: Investigation, Methodology, Validation, Writing – review & editing. CR: Investigation, Methodology, Validation, Writing – review & editing. AR: Conceptualization, Data curation, Resources, Software, Validation, Writing – review & editing. JK: Project administration, Resources, Software, Validation, Writing – review & editing. DM: Validation, Visualization, Writing – review & editing. LG: Methodology, Validation, Writing – review & editing. GB: Conceptualization, Funding acquisition, Supervision, Validation, Writing – review & editing. PC: Conceptualization, Funding acquisition, Validation, Writing – review & editing. SB: Conceptualization, Funding acquisition, Supervision, Validation, Writing – original draft, Writing – review & editing. PJ: Conceptualization, Data curation, Formal analysis, Funding acquisition, Project administration, Supervision, Writing – original draft, Writing – review & editing.
