# Supplementary material for: Associated bacterial communities, confrontation studies, and comparative genomics reveal important interactions between Morchella with Pseudomonas spp
Source: Front Fungal Biol. 2023 Dec 13;4:1285531. doi: 10.3389/ffunb.2023.1285531 (PMC10753826; doi:10.3389/ffunb.2023.1285531)
Supplement: Supplementary file 1 [file DataSheet_1.zip › Supplementary Tables and Figures.DOCX]

Associated bacterial communities, confrontation studies, and comparative genomics reveal important interactions between *Morchella* with *Pseudomonas* spp.

**Guillaume Cailleau^1^, Buck T. Hanson^2^, Melissa Cravero^1^, Sami Zhioua^1^, Patrick Hilpish^1^, Celia Ruiz^1^, Aaron Robinson^2^, Julia M. Kelliher^2^, Demosthenes Morales^3^, La Verne Gallegos-Graves^2^, Gregory Bonito^4^, Patrick S.G. Chain^2^, Saskia Bindschedler^1^, Pilar Junier^1^**

^1^Laboratory of Microbiology, University of Neuchâtel, Neuchâtel, Switzerland.

^2^Bioscience Division, Los Alamos National Laboratory, Los Alamos, NM, USA

^3^Center for Integrated Nanotechnologies, Los Alamos National Laboratory, Los Alamos, NM, USA

^4^Department of Plant, Soil and Microbial Sciences, Michigan State University, East Lansing, MI, USA

*** Correspondence:**Pilar Junier
pilar.junier@unine.ch

# Supplementary Tables

**Table S2**- Taxonomic assignment of the ASVs (provided in a separate excel file)

**Table S5**- Pairwise comparisons of *Pseudomonas* strains and ASVs (provided in a separate excel file)

**Table S1-** ITS NCBI accession numbers corresponding to the *Morchella* spp. specimens used in the analysis.

| Strain | Accession | Top_BLAST Hit | Accession Hit |
| --- | --- | --- | --- |
| M19-4 | OR482716 | *M. eximioides* | KM587917.1 |
| M19-14 | OR482775 | *M. esculenta* | MH982826.1 |
| M19-16 | OR482777 | *M. crassipes* | JQ691496.1 |
| M19-17 | OR482778 | *M. esculenta* | KM587972.1 |
| M19-18 | OR482779 | *M. esculenta* | MW307513.1 |
| M19-19 | OR482780 | *M. esculenta* | MW307512.1 |
| M19-20 | OR482781 | *M. crassipes* | JQ691496.1 |
| M19-21 | OR482782 | *M. esculenta* | MN513696.1 |
| M19-22 | OR482783 | *M. crassipes* | MT231529.1 |
| M19-23 | OR482784 | *M. esculenta* | MW307513.1 |
| M19-29 | OR482725 | *M. costata* | JQ691495.1 |
| M19-34 | OR482791 | *M. esculenta* | MW307513.1 |
| M19-35 | OR482792 | *M. esculenta* | MW307513.1 |
| M19-36 | OR482793 | *M. crassipes* | JQ691496.1 |
| M19-37 | OR482794 | *M. crassipes* | JQ691496.1 |
| M19-38 | OR482795 | *M. crassipes* | JQ691496.1 |
| M19-39 | OR482796 | *M. esculenta* | MW307513.1 |
| M19-40 | OR482797 | *M. esculenta* | KM587972.1 |
| M19-41 | OR482728 | *M. deliciosa* | MT435013.1 |
| M19-42 | OR482798 | *M. crassipes* | MT231529.1 |
| M19-43 | OR482729 | *M. angusticeps* | OK346524.1 |
| M20-7 | OR482735 | *M. elata* | KX809734.1 |

**Table S3** - Biological *Morchella* specimens used in this study. The ITS-based clade identification is provided, in addition to the sampling location (CH= Switzerland; NE= Canton of Neuchâtel; VD= Canton of Vaud) and year of obtention. Samples (Mycelium; Sclerotia) that were derived from the original fruiting body (Ascocarp) or mycelial inoculum are indicated for each specimen. The numbers indicate the number of samples. The tick marks indicate if the strains was used for the bacterial confrontation assays.

| **ID** | **Genus** | **Clade** | **Origin** | **Year of obtention** | **Bacterial community analysis** | | | **Bacterial isolation** | **Bacterial confrontation** |
| --- | --- | --- | --- | --- | --- | --- | --- | --- | --- |
|  |  |  |  |  | **Ascocarp** | **Mycelium** | **Sclerotia** |  |  |
| M19-4 | *Morchella* | Elata | CH - NE | 2019 | 1 |  |  |  |  |
| M19-14 | *Morchella* | Esculenta | CH - NE | 2019 |  | 1 |  |  |  |
| M19-16 | *Morchella* | Esculenta | CH - NE | 2019 |  | 3 | 2 |  |  |
| M19-17 | *Morchella* | Esculenta | CH - NE | 2019 | 1 |  |  |  |  |
| M19-18 | *Morchella* | Esculenta | CH - NE | 2019 |  | 1 |  |  |  |
| M19-19 | *Morchella* | Esculenta | CH - NE | 2019 |  | 1 |  |  |  |
| M19-20 | *Morchella* | Esculenta | CH - NE | 2019 |  | 1 | 1 |  |  |
| M19-21 | *Morchella* | Esculenta | CH - NE | 2019 |  | 1 |  |  |  |
| M19-22 | *Morchella* | Esculenta | CH - NE | 2019 | 1 |  |  |  |  |
| M19-23 | *Morchella* | Esculenta | CH - NE | 2019 |  | 1 | 1 |  |  |
| M19-29 | *Morchella* | Elata | CH - NE | 2019 |  | 2 |  |  | √ |
| M19-34 | *Morchella* | Esculenta | CH - NE | 2019 |  | 1 |  | 2 | √ |
| M19-35 | *Morchella* | Esculenta | CH - NE | 2019 | 1 |  |  |  |  |
| M19-36 | *Morchella* | Esculenta | CH - NE | 2019 |  | 1 | 1 |  |  |
| M19-37 | *Morchella* | Esculenta | CH - NE | 2019 |  | 1 | 1 |  |  |
| M19-38 | *Morchella* | Esculenta | CH - NE | 2019 | 1 |  |  |  |  |
| M19-39 | *Morchella* | Esculenta | CH - NE | 2019 |  | 2 |  |  |  |
| M19-40 | *Morchella* | Esculenta | CH - NE | 2019 | 1 |  |  |  |  |
| M19-41 | *Morchella* | Elata | CH - NE | 2019 |  | 3 |  |  |  |
| M19-42 | *Morchella* | Esculenta | CH - NE | 2019 |  | 3 |  |  |  |
| M19-43 | *Morchella* | Elata | CH - NE | 2019 |  | 2 |  |  |  |
| MC36 | *Morchella* | Elata | CH - NE | 2019 |  | 1 | 1 |  |  |
| MC41 | *Morchella* | Esculenta | CH - NE | 2019 | 1 |  |  |  |  |
| MC42 | *Morchella* | Esculenta | CH - NE | 2019 | 1 |  |  |  |  |
| MC43 | *Morchella* | Esculenta | CH - NE | 2019 | 1 |  |  |  |  |
| NEU142 | *Morchella* | Elata | China | 2018 |  |  | 1 |  | √ |
| NEU143 | *Morchella* | Elata | China | 2018 |  | 1 |  |  | √ |
| M84 | *Morchella* | Rufobrunnea | CH | N.A. |  |  |  | 1 | √ |
| M20-7 | *Morchella* | Elata | CH - VD-NE | 2020 |  |  |  | 3 | √ |

**Table S4** - Origin of the bacteria isolated and their *Morchella* spp. host

| Bacteria | Fungus | Origin of the fungus | Taxonomic assignment | Similarity to closest ASV |  |
| --- | --- | --- | --- | --- | --- |
| *Pseudomonas* sp. B84 | *Morchella rufobrunnea* M84 | Unknown region | *Pseudomonas baltica* | 97% (d59e483fbbe5cb7d82a87be85d1edec1) |  |
| *Pseudomonas* sp. B188 | *Morchella* sp.M19-34 | Switzerland (Neuchâtel) | *Pseudomonas putida* | 97% (71372d25e7e16dd2f95db3a9c7971517) |  |
| *Pseudomonas* sp. 33.4 | *Morchella* sp. M19-34 | Switzerland (Neuchâtel) | *Pseudomonas koreensis* | 100% (6d7c04d9e727916846e49b72f477a2f8; b60cdb3d1fc5828a04d91b26761f4a39) |  |
| *Pseudomonas* sp. VD-NE ext | *Morchella* sp. M20-7 | Switzerland (Vaud) | *Pseudomonas koreensis* | 100% (6d7c04d9e727916846e49b72f477a2f8) |  |
|  |  |  |  |  |  |
| *Pseudomonas* sp. VD-NE ins | *Morchella* sp. M20-7 | Switzerland (Vaud) | *Pseudomonas koreensis* | 100% (6d7c04d9e727916846e49b72f477a2f8) |  |
|  |  |  |  |  |  |
| *Pseudomonas* sp. VD-NE white | *Morchella* sp. M20-7 | Switzerland (Vaud) | *Pseudomonas koreensis* | 100% (6d7c04d9e727916846e49b72f477a2f8) |  |
|  |  |  |  |  |  |

# Supplementary Figures


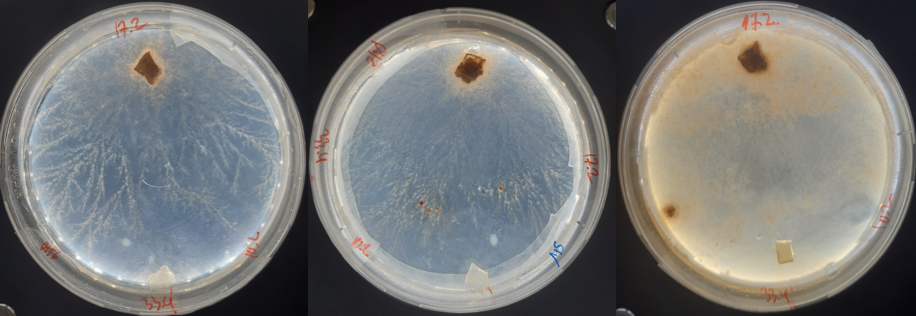


**Supplementary Figure 1.** Confrontation experiment between *Morchella* sp. strains M19-14 (top) and M19-34 (bottom) leading to the isolation of the bacterium B33.4. The bacterium was observed emerging from the mycelium of strain M19-34 when confronted with the other strain in different media.

**Supplementary Figure 2.** Number of CAZy hits in the annotated genome of the six *Pseudomonas* spp. strains.


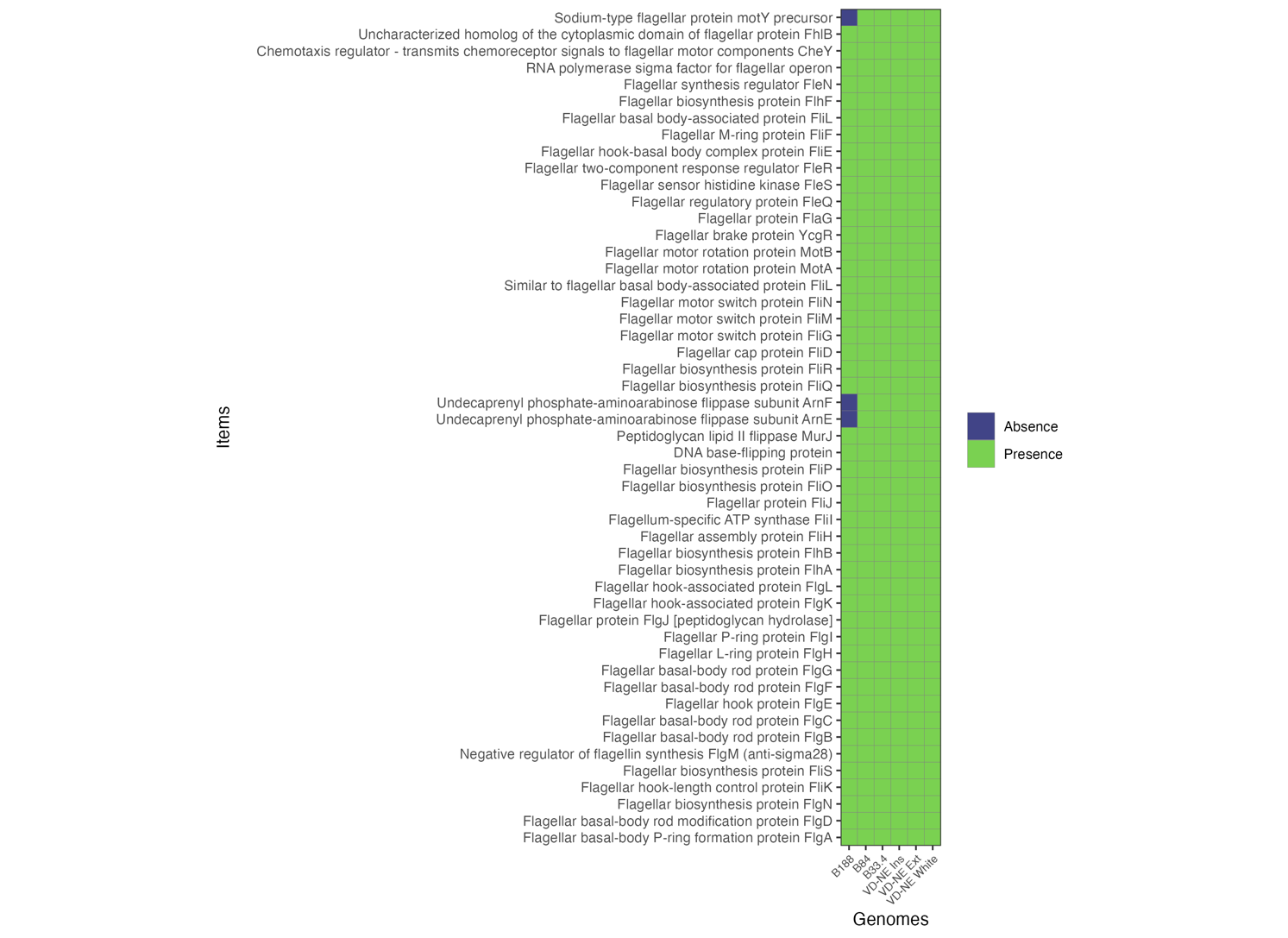


**Supplementary Figure 3.** Set of genes involved in flagellar synthesis in the genome of the six *Pseudomonas* spp. strains.
